# Lifetime cancer prevalence and life history traits in mammals

**DOI:** 10.1093/emph/eoaa015

**Published:** 2020-05-25

**Authors:** Amy M Boddy, Lisa M Abegglen, Allan P Pessier, Athena Aktipis, Joshua D Schiffman, Carlo C Maley, Carmel Witte

**Affiliations:** e1 Department of Anthropology, University of California, Santa Barbara, CA, USA; e2 Department of Pediatrics and Huntsman Cancer Institute, University of Utah, Salt Lake City, UT, USA; e3 Washington Animal Disease Diagnostic Laboratory, Washington State University, Pullman, WA, USA; e4 Department of Psychology, Arizona State University, Tempe, AZ; e5 Arizona Cancer Evolution Center, Biodesign Institute, Arizona State University, Tempe, AZ, USA; e6 Institute for Conservation Research, San Diego Zoo Global, CA, USA

**Keywords:** comparative oncology, life history theory, Peto's Paradox, cancer, mammals

## Abstract

**Background:**

Cancer is a common diagnosis in many mammalian species, yet they vary in their vulnerability to cancer. The factors driving this variation are unknown, but life history theory offers potential explanations to why cancer defense mechanisms are not equal across species.

**Methodology:**

Here we report the prevalence of neoplasia and malignancy in 37 mammalian species, representing 11 mammalian orders, using 42 years of well curated necropsy data from the San Diego Zoo and San Diego Zoo Safari Park. We collected data on life history components of these species and tested for associations between life history traits and both neoplasia and malignancy, while controlling for phylogenetic history.

**Results:**

These results support Peto’s paradox, in that we find no association between lifespan and/or body mass and the prevalence of neoplasia or malignancy. However, a positive relationship exists between litter size and prevalence of malignancy (P = 0.005, Adj. R2 = 0.212), suggesting that a species’ life history strategy may influence cancer vulnerabilities. Lastly, we tested for the relationship between placental invasiveness and malignancy. We find no evidence for an association between placental depth and malignancy prevalence (P = 0.618, Adj. R2 = 0.068).

**Conclusions:**

Life history theory offers a powerful framework to understand variation in cancer defenses across the tree of life. These findings provide insight into the relationship between life history traits and cancer vulnerabilities, which suggest a trade-off between reproduction and cancer defenses.

**Lay summary:**

Why are some mammals more vulnerable to cancer than others? We test whether life history trade-offs may explain this variation in cancer risk. Bigger, longer-lived animals do not develop more cancer compared to smaller, shorter-lived animals. However, we find a positive association between litter size and cancer prevalence in mammals.

## INTRODUCTION

Cancer is a disease that affects most multicellular organisms [1], yet we currently have a limited understanding of cancer prevalence and mortality across animals [2, 3]. Most of our cancer knowledge comes from studying humans. Comparative oncology not only increases our knowledge of cancer in animals, but it also provides new insights into cancer risk and prevention in humans. Further, quantifying cancer prevalence and cancer-related deaths in animals is important for animal health and welfare. However, previous research published on cancer in non-human animals include mostly individual case studies [4–6], which limits our ability to quantify the occurrence of cancer across animals. While some reports suggest that animals vary in susceptibility to cancer [1, 3, 7, 8], little is known about the degree of this variation. Discovering which animals are most susceptible to cancer and characterizing cancer defense mechanisms in the naturally cancer resistant animals are important next steps to understand and prevent cancer, from an evolutionary perspective.

Previous reports of cancer prevalence in wildlife [1–3, 7, 9, 10] suggest cancer susceptibility differs amongst vertebrates. The highest prevalence of cancer was reported in mammals, followed by reptiles, then birds. Amphibians had the lowest prevalence of cancer. Additionally, within mammals, cancer vulnerability ranges from relatively cancer-free species, such as the naked-mole rat [11] and blind mole rat [12], to cancer-prone species, such as dogs [13], ferrets [14] and Tasmanian devils [15]. A life history theory (LHT) framework can help explain this variance in cancer rates across animals [16]. LHT is an evolutionary and ecological approach that investigates organism-level trade-offs between growth, maintenance and reproduction. According to LHT, long-lived animals invest more energy in somatic maintenance (e.g. cancer defenses) to maintain their cellular bodies for decades [16], whereas short-lived animals invest more of their resources in reproduction to produce more offspring in short periods of time. Here, we test whether life history traits of body mass, lifespan and reproduction (e.g. litter size) predict cancer prevalence across mammals.

Consistent with LHT predictions, Peto’s Paradox is the observation that larger, longer-lived animals do not develop more cancer compared with smaller, shorter-lived animals, despite the fact that large, long-lived animals have more cells with more opportunities to accumulate cancer causing mutations [17–19]. Indeed, Abegglen *et al.* [2] reported the first empirical evidence for Peto’s Paradox by analyzing cancer prevalence in 37 mammals. This study suggests that larger, longer-lived animals have enhanced cancer defense mechanisms. Additionally, extra copies of *TP53*, a critical tumor suppressor gene, were reported in elephants, and functional studies identified this gene expansion as a potential mechanism of cancer defense in the largest extant land mammal.

Along with body size and lifespan as predictors of cancer mortality, placental mammals may have higher rates of malignancy due to selection for invasive placental genes [20, 21]. Placentation and embryo implantation share similar biological processes to malignancy, including tissue invasion, extracellular matrix degradation, angiogenesis initiation, cellular migration and maternal immune system evasion [21]. These mechanisms of placentation may be co-opted by cancer cells during neoplastic progression. Additionally, the depth of placentation varies among mammals. Some evidence suggests that the degree of placental invasiveness correlates with malignancy prevalence in certain mammals [21]. We predicted that species with the most invasive placenta type (hemochorial) would have higher rates of malignancy compared with animals with less invasive placentas (endotheliochorial and epitheliochorial).

In this study, we retested Peto’s Paradox to answer the question: Do larger, long-lived mammals get more cancer? We then analyzed the association between cancer risk and life history traits in a phylogenetic context, including the degree of placental invasiveness. Lastly, we show how working together to combine an evolutionary approach with the knowledge, resources and expertise of animal health experts can help explain how species across the tree of life have dealt with cancer as a selective pressure.

## METHODS

### Data collection

Building on data previously reported in the Abegglen 2015 study, cancer prevalence data were re-abstracted from Griner [22]. This book published necropsy findings from animals housed in the San Diego Zoo and San Diego Zoo Safari Park, collectively referred to as San Diego Zoo Global (SDZG). The necropsies were reported from 1964 to 1978. We combined this data with recent prevalence estimates based on mortality records from SDZG, which were extracted from SDZG electronic records collected from 1987 through 2015. We note these current data (1987–2015) were collected during a period when complete post-mortem examinations and histopathology were performed on complete tissues representing all major organ systems. Histopathology was performed on cases compiled by Griner as well, but necropsy protocols were less standardized during that time (1964–78).

All data analyzed herein were summarized and interpreted by a board-certified veterinary pathologist and epidemiologist from SDZG. We report mortality records with attention to important interpretation details that were not available in the original report by Abegglen *et al.* [2] (Supplementary Table S1). Data were filtered to exclude stillbirths, perinatal mortalities and animals less than 1 year of age with a low risk for developing cancer. Exclusion of these individuals decreases potential bias that would result in lower estimated cancer prevalence rates.

We then refined the case definition of neoplasia to distinguish benign vs malignant neoplasms. A neoplasm is a general term for an abnormal growth that includes both benign and malignant tumors. We removed hyperplastic foci in thyroids from the neoplastic category for the Tasmanian devil (see additional methodological details in footnotes of Supplementary Table S1). While all cancers are not the same, and combining all cancers may be considered crude, it illustrates the importance of refining and standardizing definitions of neoplasia in comparative mortality studies. For data summarized from contemporary records (1987–2015 dataset), we listed specific neoplastic and malignant conditions observed (Supplementary Table S1). The type of cancer or malignancy was not always specified in historic data reported by Griner [19]. The final dataset includes 42 years of data on 852 necropsies representing 37 mammals in 11 mammalian Orders (Supplementary Table S1).

### Estimates of cancer prevalence

All neoplasia diagnoses, both benign and malignant were recorded (numerator for prevalence estimates), as well as the total number of individuals necropsied (denominator for prevalence estimates)—allowing us to estimate disease prevalence. Lifetime prevalence of neoplasia and malignancy (number of cases of cancer in necropsies/number of total necropsies) was determined for species where at least 10 individuals with necropsies were available to meet the inclusion criteria for the at-risk population. Due to small post-mortem samples sizes, Asian and African elephant neoplasia and malignancy data were combined. Confidence intervals (95%) on lifetime neoplasia prevalence were estimated in PropCI package in R.

### Life history regression models

We tested for a relationship between life history variables and cancer prevalence. Life history variables were collected from Pantheria [23] and AnAge [24]. Information on placental types was compiled from published sources [25, 26]. While we used an estimated lifespan in elephants to be 65 years, we note that maximum lifespan in Asian elephant may be as high as 80 years [27].

To test for the relationship between life history variables (body mass, lifespan, litter size and placenta invasiveness) and either neoplasia or malignancy, we used phylogenetic linear regression (PGLS) models. Conventional statistical methods fail to account for patterns of phylogenetic relatedness among organisms due to evolutionary history [28]. Accordingly, we tested for associations in our data by implementing a PGLS model that corrected for non-independence due to common ancestry in the species. In the PGLS models, we used maximum likelihood (ML) estimation of model parameters to account for species’ shared ancestry [29]. We used the R package CAPER [30] and the updated mammalian super-tree [31]. Estimations of phylogenetic signal (*λ* parameter) were performed using CAPER. Species data points varied in the number of animals necropsied. To control for this variability, we used total animals necropsied as a covariate in the PGLS multiple regression model. We estimated Akaike information criterion (AIC) for our LH models, and report models with the best fit, using the criteria of ΔAIC > 2 as substantial evidence for model fit (Supplementary Table S3). To identify phylogenetic outliers in the PGLS model, we extracted phylogenetic residuals in CAPER. To test for a relationship between placental invasiveness and malignancy, we created dummy variables to represent degree of placentation, setting marsupials as the reference level. Similar methods were previously used for testing relationships between placental morphology and life history traits [32, 33].

## RESULTS

**Figure 1.**
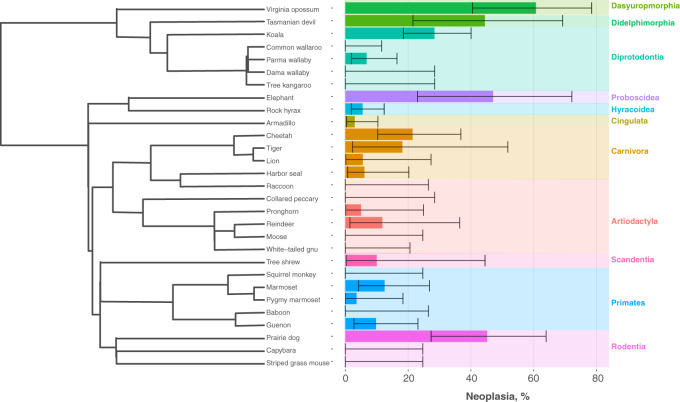
Lifetime neoplasia prevalence in 29 mammals. Bar plots representing the neoplasia prevalence for mammals . We estimated prevalence and 95% confidence intervals (CI) for mammals that had *n* ≥ 10 individuals per species (*n* = 29). Error bars indicated 95% CI. All data for neoplasia and malignancy for the full 37 species are in Supplementary Tables S1 and S2. Species were organized according to their phylogenetic relationships, in which we saw no clear patterns across the mammalian orders.

Here, we report on neoplasia and malignancy prevalence in 37 mammals in a highly curated post-mortem dataset from SDZG. Of the 852 necropsies in this dataset, we report 112 records of neoplasia and 83 records of malignancy. Cancer prevalence varied substantially across taxa (*n* = 29 species with at least 10 necropsies to estimate prevalence). Neoplasia prevalence ranged from 0–60.7% (Fig. 1), with an estimated mean of 12.5%. Malignancy prevalence ranged from 0–54%, with a mean of 9% (Supplementary Table S1).

In our dataset, the Virginia opossum, *Didelphis virginiana* had the highest prevalence of neoplasia (*n* = 17/28; 60.7%). The animal with the second highest prevalence of neoplasia was the Prairie dog, *Cynomys ludovicianus* (*n* = 14/31; 45%). In contrast, we found species with no reports of neoplasia and/or malignancy, including two species from the Order Artiodactyla; Moose, *Alces alces*, (*n* = 0/13), and White-tailed gnu, *Connochaetes gnou*, (*n* = 0/16). Interestingly, Armadillos, *Dasypus novemcinctus*, had no reports of malignancy in 67 necropsies. Though Tasmanian Devils have a relatively high prevalence of neoplasia (44%, 8 of 18), because these animals were housed within a zoo, there are no transmissible facial tumors in our dataset [15].

**Figure 2.**
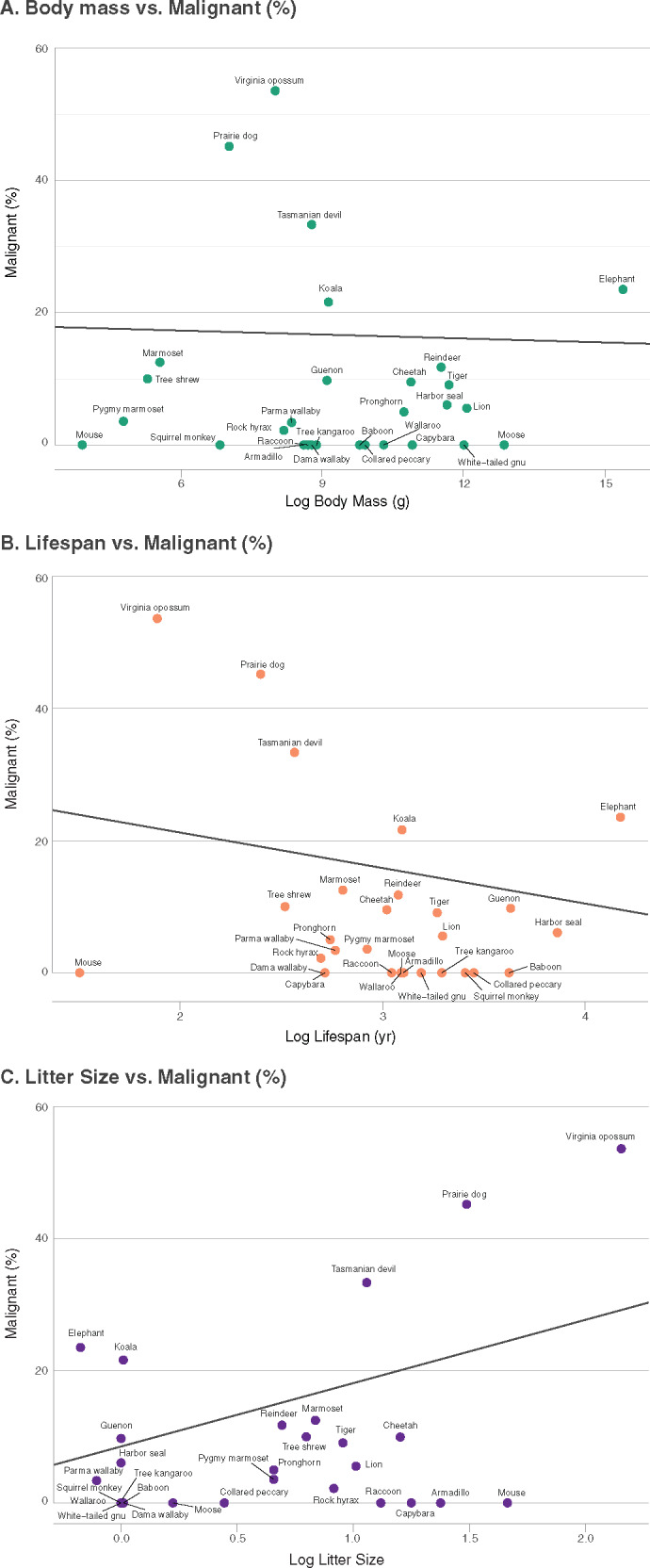
Relationship between malignancy and life history traits in mammals. Percentage of malignancy in 29 species, representing 800 necropsies, of mammals in relation to three life history traits: (**A**) body mass (g); (**B**) lifespan (years) and (**C**) litter size. We used a phylogenetic comparative method to determine the association between life history traits and malignancy. The black line represents the phylogenetic comparative method generalized least squares (PGLS) regression model.

**Figure 3.**
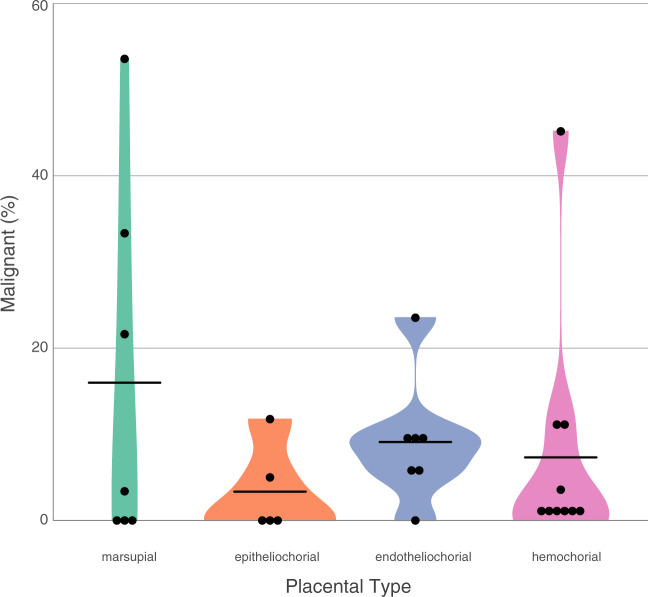
Relationship between malignancy and the degree of placentation in mammals. Mammalian placentas can be classified on the degree of invasiveness. Here we plotted the relationship between malignancy and placenta invasiveness. Degree of placentation was grouped from left to right, with marsupials on the far left representing rudimentary yolk-sac placentas, then Eutherian placenta classifications: epitheliochorial (least invasive), endotheliochorial (intermediate invasive) and hemochorial (most invasive). We found no association between degree of placentation and malignancy or neoplasia (also see Supplementary Fig. S5).

We then tested for the relationship between life history characteristics and neoplasia and/or malignancy prevalence, while incorporating phylogenetic history into the model (Fig. 2). We tested for non-independence in our dataset and found phylogenetic signal estimates were high (*λ* ∼1), demonstrating species within our dataset resemble each other more than species drawn at random. We report no phylogenetic outliers in the dataset. Of the 29 mammals analyzed (800 necropsies), our dataset varied in body mass and lifespan (Supplementary Table S2). The smallest animal was the striped grass mouse (0.05 kg, maximum lifespan 4.5 years) and the largest animal was the elephant (4800 kg, maximum lifespan 65 years). In support of Peto’s Paradox, cancer prevalence did not increase with body mass or maximum lifespan (Table 1A). Litter sizes ranged from singleton births (e.g. elephants) to approximately eight offspring (e.g. opossum). We found a significant relationship with litter size and neoplasia prevalence (*t *=* *2.736, *P *=* *0.01) and malignancy (*t *=* *3.081, *P *=* *0.005) (Table 1A). However, this relationship only trended toward significance when we removed the Virginia opossum from the analyses (Supplementary Table S3). Our dataset had representation from all three invasive placental types epitheliochorial (*n* = 5), endotheliochorial (*n* = 7) and hemochorial (*n* = 10), and data from marsupials (*n* = 7). Malignancy prevalence had no significant relationship with degree of placentation (Fig. 3, Table 1B).

**Table 1.** PGLS models

Life history predictors	*t*-Value	*P*-Value	ML lambda	Adjusted *R*^2^
(A) PGLS malignant prevalence and LH models summary, *n* = 29 species				
Body mass	−0.176	0.862	0.99	0.001
Lifespan	−1.455	0.157	1	0.005
Litter size	3.081	0.005	0.99	0.212
(B) PGLS malignant prevalence and placenta model summary, *n* = 29 species				
Placental invasiveness	*t*-Value	*P*-Value	ML lambda	Adjusted *R*^2^
Intercept	1.788	0.086	1	0.068
Epitheliochorial	−1.226	0.232		
Endotheliochorial	−0.928	0.3625		
Hemochorial	−1.188	0.2463		

Here, we report the summary of the PGLS models of malignancy prevalence in 29 species and 800 individual necropsies. (A) PGLS models testing the relationship between malignancy and three life history traits: body mass, lifespan and litter size. Body mass was controlled for in the lifespan and litter size models reported here. Neoplasia models are reported in Supplementary Table S3. (B) A PGLS model testing the relationship between placental invasiveness and malignancy, using placenta, dummy coded, following [31, 32]. For models A and B, we report the *t*-value and *P*-value. We also report lambda, the estimated measure of phylogenetic signal.

## DISCUSSION

Species vary predictably across a number of parameters (body size, longevity, reproductive effort). We hypothesized that life history measures influence cancer suppression mechanisms and should, therefore, be associated with the risk of developing malignancy. To test these predictions, we modeled the relationship between life history factors (body mass, lifespan, litter size and placental invasiveness) with neoplasia and malignancy prevalence in 29 mammalian species. Consistent with previous reports on Peto’s Paradox [2], we found no relationship between neoplasia/malignancy and body mass and/or lifespan, even when phylogenetic history was included in the model. However, we did find a significant relationship with malignancy prevalence and litter size in mammals. Lastly, we found no relationship between placental invasiveness and malignancy.

Sir Richard Peto’s observation was paradoxical from a cancer biology perspective: because every cell in a body has a chance of becoming cancerous, due to the accumulation of cancer causing mutations, organisms with more cells, maintained for longer periods of time, should develop more cancer [17, 18, 34]. However, Peto’s observation was not paradoxical from an evolutionary point of view. LHT suggests that large, long-lived organisms experience the most selective pressure to evolve cancer defenses [16, 35]. This selective pressure leads to an important implication for evolutionary medicine. Large and/or long-lived organisms may hold biological secrets to novel cancer defense mechanisms. Large body size and long lifespan evolved independently many times across the tree of life, suggesting that numerous mechanisms of cancer prevention await discovery [36, 37].

Zoological data are a critical resource for comparative oncology and evolutionary medicine. However, collecting reliable data on cancer prevalence in non-human animals has been challenging. Even within a single institution, not all animals receive a necropsy and recording methods often are not consistent. Here we benefited from decades of careful practice and recording at SDZG, including expert veterinary pathologists who were available on site to interpret and classify the results. Our results are consistent with previously published case studies. For example, we found the most commonly diagnosed neoplastic condition in Virginia opossums was bronchoalveolar carcinoma (53%; 8 out of 15 diagnosed neoplasia). Bronchoalveolar carcinomas were reported previously in Virginia opossums, but never quantified [38]. Also in agreement with our data, 30% neoplasia prevalence was previously reported in black-footed prairie dogs (50/167 animals) [39]. We observed 45% neoplasia prevalence in our dataset (14/31 animals). The difference in prevalence suggests our current estimate for the black-footed prairie dogs and other animals may be noisy, due to our smaller sample sizes. Additionally, we find no reports of neoplasia or malignancy in moose and white-tailed gnus, large-bodied species of the Artiodactyla order. We also observed no cancer in armadillos. These results warrant further investigation to determine if armadillos and other large-bodied Artiodactyla are better at suppressing cancer compared with other species. Lastly, this report highlights the utility of well curated cancer across species data and provides exciting new opportunities for cancer comparative genomics and biology research.

### The elephant in the room

We report higher cancer prevalence in elephants than previously reported [2]. Previous estimates were derived from the Elephant Encyclopedia Database (*n* = 644 elephants) [2]. While this database is an important resource for the elephant community, we are not confident that all of the data were medically curated. Our current data, from a medically curated database, were reviewed by a board-certified veterinary pathologist. Standardized disease surveillance was performed on all animals that died. Importantly, we were able to differentiate between neoplasia and malignancy. Malignancy was found in 4 out of 17 elephant necropsies (24% prevalence of malignancy, 95% CI: 7–50%). Of the malignant tumors reported, two malignancies were found in the uterus (1 uterine adenocarcinoma and 1 undifferentiated uterine malignant neoplasm), one leiomyosarcoma was found in the lung and one sarcoma was found in the liver.

These results demonstrate a greater need for collaborations with zoological institutions and a need for well-developed pathology programs with long-term medical data across zoological institutions. The higher prevalence of neoplasia in elephants reported here will not be a surprise to the elephant veterinary community, as it is common knowledge that older female elephants develop uterine lesions [40]. Many of observed lesions are benign growths or leiomyomas (fibroids), similar to the benign uterine fibroids that occur in over 70% of women throughout their lifetime [41].

Other discrepancies in our dataset compared with the Abegglen 2015 study include differences in data interpretation (e.g. at-risk groups, refining the case definition) and new trends in prevalence. For example, our dataset showed an increase in koala neoplasia prevalence from 3.8% [2] to 42% (*n* = 20/48, including 15 malignant). This increase in koala neoplasia prevalence is likely due to the Koala retrovirus (KoRV) that was discovered in 2006 [42]. These patterns demonstrate the importance of collecting data over time, which can highlight emerging infections or environmental changes that may require increased monitoring for cancer.

### Placenta invasiveness is not a predictor of cancer risk

Mechanisms of placentation are similar to the hallmarks of cancer, which include growth, invasion, vascularization and immune modulation [20, 21]. A previous study reported that malignancy risk co-varied with the depth of placentation in four mammals, yet, this study only represented two of the least invasive placenta types, epitheliochorial and endotheliochorial [21]. Indeed, when we restricted our data to include only those four mammals (Fig. 3), we also observed greater malignant prevalence in the more invasive endotheliochorial placenta type (9.1% mean malignancy) than epitheliochorial (3.4% mean malignancy). However, when we analyzed our full dataset of 29 mammals representing all categories of placentation, we found no relationship between the *degree* of placentation and cancer malignancy.

Why may the degree of placentation have no relationship with malignancy? The placenta is the site of intense evolutionary conflict between maternal–paternal genes [20, 43, 44]. As a consequence of this evolutionary conflict, the placenta is one of the most diverse mammalian organs. Across various mammalian species, placenta evolved different degrees of invasion, including multiple independent reductions in invasiveness over evolutionary time [45, 46]. Indeed, malignancy risk may not be generalizable and highly invasive placental mammals (hemochorial) may have co-evolved heighten strategies to defend against inappropriate invasion mechanisms often co-opted by cancer cells. In addition, mammalian placenta differs in surface area morphology (i.e. degree of interdigitation) across species. Future studies are needed to test the relationship between cancer risk and placenta morphology and/or placenta interdigitation [47].

### Quality not quantity may predict cancer prevalence

According to LHT, species may evolve traits that increase their reproductive success at the cost of somatic maintenance, which will affect their cancer defenses [16, 34, 35]. As predicted, we found a positive relationship between litter size and frequency of neoplasia and malignancy. While part of this relationship was driven by an outlier, the Virginia opossum, this relationship is worthy of further investigations that include more animals with large litter sizes. Indeed, reproductive output (i.e. litter size) and cancer risk could be a case of antagonistic pleiotropy [48], where selection on phenotypes that benefit a species early in life, may have deleterious effects later in life. There may be alleles with pleiotropic effects on both litter size and cancer susceptibility. Interestingly, the estrogen receptor (ER) locus and the gonadotropin-releasing hormone receptor (GnRHR) are associated with litter size in domestic farm animals [49, 50] and also expressed in many tumor types [51, 52].

Litter size may not be directly related to cancer susceptibility, but it may be a good indicator of life history strategy. This is because species life history traits are tightly correlated. Fast life history organisms, characterized by large litter sizes and short lifespans [53], tend to invest in offspring quantity over offspring quality. Offspring quality is likely associated with somatic maintenance (e.g. cancer defenses via DNA repair or immune surveillance). Interestingly, neoplasia is rarely reported in naked mole rats, [8, 54] but they have large litter sizes (e.g. 3–12 offspring) [8]. Naked mole rats also have a unique life history strategy. They are the only eusocial mammal, in which only the queen produces litters. In addition, they have very long lifespans for mammals of their body size [55].

### Study limitations

Similar to humans, cancer rates in animals likely vary by age, sex and other demographic factors. Our estimates are from sample sizes, which lead to wide confidence intervals. However, these data provide an important starting point for quantifying lifetime cancer prevalence across broad taxonomic groups. The advantages of our data include complete post-mortem surveillance on all animals that died at these institutions from 1987 to 2015. Histopathology was performed on only select tissues prior to 1987. Post-mortem exams earlier than 1987 could have missed early-stage lesions. However, it is unlikely that larger, more advanced lesions were missed. Every animal included in our analyses had post-mortem examinations carried out by pathologists.

We acknowledge that cancer is not a single disease. However, in this initial study, we combined all cancer types, due small sample sizes and the lack of standardized documentation of cancer types across all historic records. Despite this limitation, we hope this study highlights the need for standardization of health record terminology and documentation in both animal and human medical settings. Data harmonization will help to move the field of cancer biology forward and unite human and veterinarian medicine. In the future, refinement of age and tissue-specific cancer prevalence estimates at the taxonomic level over time could be achieved through cross-institution collaborative efforts to compile post-mortem findings.

Lastly, we recognize that managed populations may have different exposures and protective factors compared with free ranging wildlife, and that they live in different environments than they evolved. Here we have only measured cancer prevalence in managed environments. The average lifespan of mammals from housed/managed populations tends to be longer than free-ranging wildlife. This increased lifespan is most notable in animals with short lifespans in the wild due to predation, intra-specific competition and disease [56]. Our results likely are biased toward increased cancer prevalence in managed populations compared with wild populations, because cancer is a disease of aging populations. Further age-adjustment would provide a clearer picture of cancer resistance and can identify species with low cancer rates in the highest risk, geriatric groups.

## CONCLUSIONS

Mammals vary in cancer vulnerabilities. Here, we provide a highly curated and expanded dataset on cancer prevalence in 37 mammalian species. We hypothesized that several important life history features explain variation in cancer across mammals. We found no relationship between cancer prevalence and longevity and/or body size, i.e. Peto’s paradox holds across mammals. However, we found a significant positive relationship between litter size and cancer prevalence. In contradiction to our predictions, we found no relationship between placentation depth and malignancy. This study shows how a comparative approach to cancer research can help us discover distinct anti-cancer adaptations in particular taxa, and provide new insights into cancer prevention and clinical management of both human and animal cancers.

## SUPPLEMENTAL MATERIAL

Supplementary data is available at *EMPH* online.

## Supplementary Material

eoaa015_Supplementary_Data
