# Supplementary material for: Lifetime cancer prevalence and life history traits in mammals
Source: Evol Med Public Health. 2020 May 25;2020(1):187–95. doi: 10.1093/emph/eoaa015 (PMC7652303; doi:10.1093/emph/eoaa015)
Supplement: eoaa015_Supplementary_Data [file eoaa015_supplementary_data.zip › Sup_figs.docx]

Supplemental Figures:

**Figure S1: Ancestral Reconstructions of Malignancy Prevalence in Mammals**.

Here we used the R package PHYtools to reconstruct ancestral state of malignancy in these 29 mammals. Phylogenetic tree pruned from updated mammalian super-tree published by Bininda-Emonds in 2007.

**Figures S2-4: Relationship between neoplasia and life history traits in mammals**

Percentage of malignancy in 29 species of mammals in relation to three life history traits: body mass (g), lifespan (years)and litter size. We used phylogenetic comparative methods to determine the relationship between life history traits and neoplasia. Black line represents the phylogenetic comparative methods generalized least squares (PGLS) regression model. Blue line represents linear regression model without controlling for phylogeny. Size of the dots represents the total number of individuals necropsied for that species.

**S2: Relationship neoplasia and litter size**

**S3: Relationship neoplasia and lifespan**

**S4: Relationship neoplasia and body mass**

**S5: Relationship between the degree of placentation and neoplasia in mammals**

Mammalian placentas can be classified on the degree of invasiveness. Here we plotted the relationship between neoplasia and placenta invasiveness. Degree of placentation was grouped from left to right, with marsupials on the far left representing rudimentary yolk-sac placentas, then eutherian placenta classifications: epitheliochorial (least invasive), endotheliochorial (intermediate invasive), and hemochorial (most invasive). We found no relationship between degree of placentation and malignancy or neoplasia (also see Fig 3). Size of the dots represents the total number of individuals necropsied for that species.
